# Phosphatidylinositol(4,5)bisphosphate: diverse functions at the plasma membrane

**DOI:** 10.1042/EBC20200041

**Published:** 2020-08-26

**Authors:** Matilda Katan, Shamshad Cockcroft

**Affiliations:** 1 Institute of Structural and Molecular Biology, Division of Biosciences, University College London, Gower Street, London WC1E 6BT, U.K.; 2 Department of Neuroscience, Physiology and Pharmacology, Division of Biosciences, University College London, 21 University Street, London WC1E 6JJ, U.K.

**Keywords:** endocytosis, exocytosis, phosphatidylinositol, phospholipases

## Abstract

Phosphatidylinositol(4,5) bisphosphate (PI(4,5)P_2_) has become a major focus in biochemistry, cell biology and physiology owing to its diverse functions at the plasma membrane. As a result, the functions of PI(4,5)P_2_ can be explored in two separate and distinct roles – as a substrate for phospholipase C (PLC) and phosphoinositide 3-kinase (PI3K) and as a primary messenger, each having unique properties. Thus PI(4,5)P_2_ makes contributions in both signal transduction and cellular processes including actin cytoskeleton dynamics, membrane dynamics and ion channel regulation. Signalling through plasma membrane G-protein coupled receptors (GPCRs), receptor tyrosine kinases (RTKs) and immune receptors all use PI(4,5)P_2_ as a substrate to make second messengers. Activation of PI3K generates PI(3,4,5)P_3_ (phosphatidylinositol(3,4,5)trisphosphate), a lipid that recruits a plethora of proteins with pleckstrin homology (PH) domains to the plasma membrane to regulate multiple aspects of cellular function. In contrast, PLC activation results in the hydrolysis of PI(4,5)P_2_ to generate the second messengers, diacylglycerol (DAG), an activator of protein kinase C and inositol(1,4,5)trisphosphate (IP_3_/I(1,4,5)P_3_) which facilitates an increase in intracellular Ca^2+^. Decreases in PI(4,5)P_2_ by PLC also impact on functions that are dependent on the intact lipid and therefore endocytosis, actin dynamics and ion channel regulation are subject to control. Spatial organisation of PI(4,5)P_2_ in nanodomains at the membrane allows for these multiple processes to occur concurrently.

## Introduction

**Figure 1:**
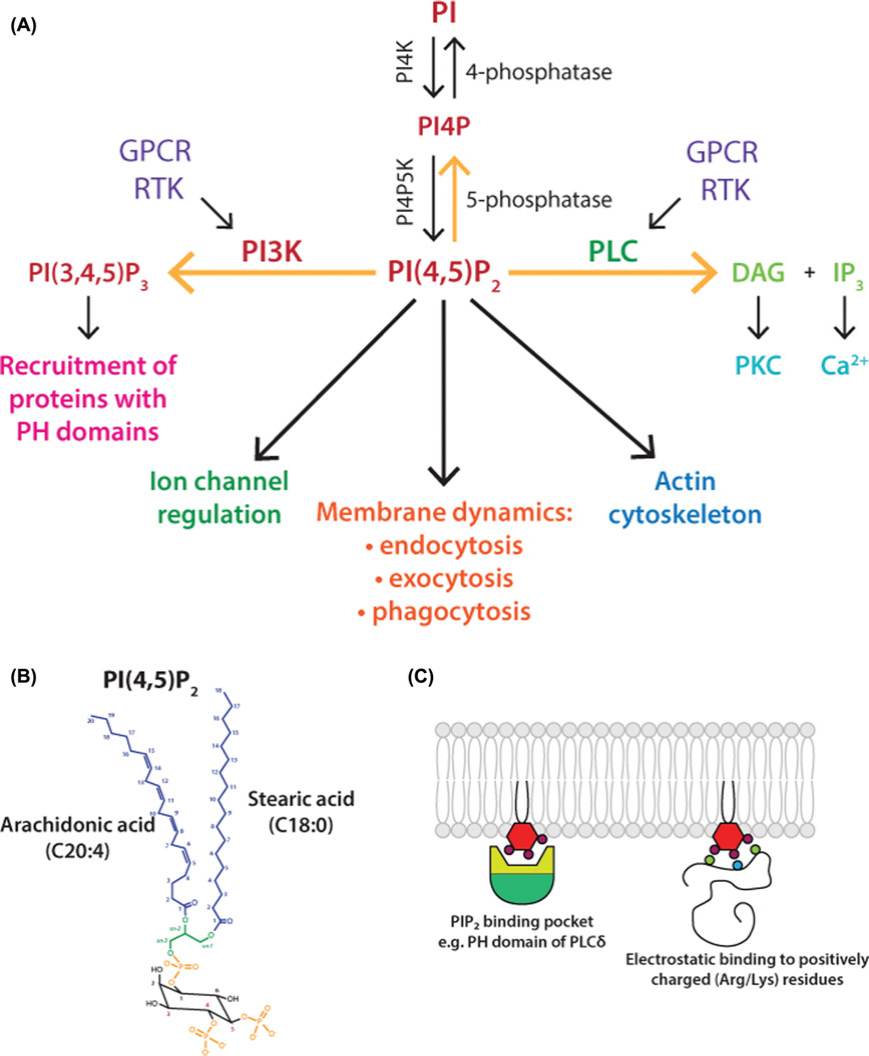
Phosphatidylinositol(4,5)bisphosphate (PI(4,5)P_2_) (**A**) **Multiple functions of PI(4,5)P_2_ at the plasma membrane**. PI(4,5)P_2_ is a substrate for two signalling pathways, phospholipase C (PLC) and phosphoinositide 3-kinase (PI3K). PI(4,5)P_2_ also functions as an intact lipid to regulate ion channels, membrane dynamics and the actin cytoskeleton. Three pathways can deplete PI(4,5)P_2_ levels, marked with yellow arrows – PLC, PI3K and 5-phosphatase. Abbreviations: DAG, diacylglycerol; GPCR, G-protein coupled receptor; IP_3_, inositol(1,4,5)trisphosphate; PI, phosphatidylinositol; PI4K, PI 4-kinase; PI4P, phosphatidylinositol 4-phosphate; PI4P5K, PI4P 5-kinase; PKC, protein kinase C; RTK, receptor tyrosine kinase. (**B**) **Structure of PI(4,5)P_2_**. PI(4,5)P_2_ comprises a glycerol backbone with an inositol headgroup which is phosphorylated at the 4 and 5 positions on the inositol ring. The fatty acid composition of PI(4,5)P_2_ is distinctive; stearic acid (C18:0) at the *sn*-1 position and arachidonic acid (C20:4) at the *sn*-2 position of the glycerol backbone. (**C**) **PI(4,5)P_2_ can bind domains such as PH or by electrostatic interactions to basic residues of arginines and lysines**. PI(4,5)P_2_ can bind to structured domains such as PH domains or it can bind to unstructured clusters of positively charged lysine and arginine residues in proteins due to electrostatic interactions. Abbreviation: PH domain, pleckstrin homology domain.

Phosphatidylinositol(4,5)bisphosphate (PI(4,5)P_2_), is a low abundance, cellular membrane phospholipid generated by phosphorylation of phosphatidylinositol (PI) (Figure 1A). PI, the parent lipid of all phosphoinositides, comprises between 5 and 8% of the total lipids of the cell [1]. The inositol head group can be reversibly phosphorylated at 3, 4 and 5 positions giving rise to seven phosphoinositide derivatives. Approximately, 10% of the PI is in the phosphorylated state [2]. These minor phosphorylated derivatives (PI4P, PI3P, PI5P, PI(4,5)P_2_, PI(3,4)P_2_, PI(3,5)P_2_ and PI(3,4,5)P_3_ (phosphatidylinositol(3,4,5)trisphosphate)) are distributed in different membrane compartments determined by the presence of the kinases that phosphorylate the inositol ring. Of these, PI(4,5)P_2_ is the most abundant phosphoinositide and is enriched in the cytoplasmic leaflet of the plasma membrane comprising 1–2 mol% of total plasma membrane lipid [3,4]. One of the most striking characteristics of mammalian PI and its derivatives is its acyl chain composition. The fatty acids linked to the glycerol backbone are predominantly, stearic acid (C18:0; 18 carbons with no double bonds) at the *sn-1* position and arachidonic acid (C20:4; 20 carbons with 4 double bonds) at *sn-2* position [5,6] (Figure 1B).

PI(4,5)P_2_ regulates many aspects of cell function at the plasma membrane (Figure 1A) (reviewed in [7–17]). PI(4,5)P_2_ is a substrate for two signalling pathways. Phospholipase C (PLC) leads to the generation of two second messengers, inositol(1,4,5)trisphosphate (I(1,4,5)P_3_), known trigger for mobilising Ca^2+^ from endoplasmic reticulum (ER) stores and diacylglycerol (DAG), an activator for protein kinase C (PKC). The second pathway is phosphoinositide-3-kinase (PI3K) to make PI(3,4,5)P_3_, a lipid with wide-ranging functions. Thus, PLC, PI3K, PI4P 5-kinases (PI4P5Ks) and PI(4,5)P_2_ 5-phosphatases maintain dynamic turnover and tight spatiotemporal control of PI(4,5)P_2_ levels (Figure 1A). This is important as PI(4,5)P_2_ as an intact lipid regulates diverse cellular functions, including cytoskeletal organisation and membrane trafficking (including endocytosis and exocytosis) and ion channel regulation [10–13]. PI(4,5)P_2_ interacts with a variety of binding proteins including ANTH (AP180 N-Terminal Homology), ENTH (Epsin N-Terminal Homology), C2 (protein kinase C conserved region 2), FERM (a domain named after four proteins, Band 4.1, ezrin, radixin and moesin), PDZ (named after three proteins, PSD95, Dig1 and Zo-1 that share the domain), PH (pleckstrin homology) and Tubby domains (Tubby domain first identified in the Tubby protein) [8,18], indicating diverse downstream effectors of PI(4,5)P_2_. In addition, PI(4,5)P_2_ is a highly negatively charged lipid and therefore can bind unstructured clusters of basic residues on numerous membrane proteins (for example, ion channels, receptors and cytoskeletal proteins) [3,11] (see Figure 1C). Functions of PI(4,5)P_2_ are prolific due to the large number of effector proteins identified as PI(4,5)P_2_ binding proteins and Table 1 provides examples of PI(4,5)P_2_ functions at the plasma membrane.

**Table 1:** Summary of milestones in the field

PI(4,5)P_2_-dependent functions at the plasma membrane	Comments
Substrate for PLC to make second messengers, I(1,4,5)P_3_ and DAG	This lipid signalling pathway was first described in 1953 [139]; it was only in 1983 that the second messengers and their functions were discovered [140].
Regulation of the actin cytoskeleton by PI(4,5)P_2_ [99]	The first two actin-binding proteins identified to interact with PI(4,5)P_2_ were profilin in 1985 [141] and gelsolin in 1987 [142]. Many actin-regulatory proteins are activated or inactivated by binding to PI(4,5)P_2_ [14,91].
Substrate for PI3-kinase to make PI(3,4,5)P_3_; the lipid recruits a subset of PH domain-containing proteins including AKT	This pathway was discovered in 1988 [143,144]; insulin-mediated signalling utilises this pathway for glucose uptake [67].
The PH domain of pleckstrin was first shown to bind specifically to PI(4,5)P_2_	PH domains are 120 amino acids in length, the first PH domain was detected in pleckstrin in 1994, hence the domain name [145]. The PH domain of PLCδ1 binds to PI(4,5)P_2_ with high affinity and the GFP (green fluorescent protein)-fusion protein is used to monitor PIP(4,5)P_2_ in living cells.
Exocytosis: mediates release of hormones, neurotransmitters from neurons and neuroendocrine cells. PI(4,5)P_2_ is required for priming and for exocytic fusion [111]	Priming factor, CAPS (Ca^2+^-dependent activator protein for secretion), recruited by PI(4,5)P_2_ was the first protein identified in 1992 [146,147]; Syntaxin 1 clustered at the plasma membrane by PI(4,5)P_2_ [110]; Synaptagmin-1 and Doc2β are recruited to plasma membranes by PI(4,5)P_2_ and are essential for exocytosis [109,148].
Ion channels and transporters – PI(4,5)P_2_ have multiple effects dependent on the ion channels and transporters [112]	The first paper to implicate PI(4,5)P_2_ in regulation of the Na^+^/Ca^2+^ exchanger and K_ATP_ channels was published in 1996 [149]. Kir (inward rectifying K^+^) channels are maintained in the open state by PI(4,5)P_2_; hydrolysis of PI(4,5)P_2_ by PLC closes the channels [12]; KCNQ (Kv7) are voltage-gated channels and PI(4,5)P_2_ up-regulates both the current amplitude and voltage sensitivity of the KCNQ2 channel. Disruption of the interaction of PI(4,5)P_2_ with the S4–S5 linker of KCNQ by a single mutation decreases the voltage sensitivity and current amplitude [150].
Clathrin-mediated endocytosis: PI(4,5)P_2_ is required for AP2 binding to membranes	The first paper identifying PI(4,5)P_2_ for recruitment of AP2 was first published in 1998 [151]. Another protein that is recruited by PI(4,5)P_2_ is dynamin, and was first shown in 1996 [99,152,153].
GPCRS have hot spots for PI(4,5)P_2_ and can form bridging interactions with Gα subunits or with arrestin	The first paper to identify PI(4,5)P_2_ binding to GPCRS was published in 2018 [119]. β1 adrenergic receptors–Gαs interaction is stabilised by the binding of two molecules of PI(4,5)P_2_ [119]; phosphorylated neurotensin receptor 1 bound to arrestin is bridged by one molecule of PI(4,5)P_2_ [121].

## Synthesis of PI(4,5)P_2_

**Figure 2:**
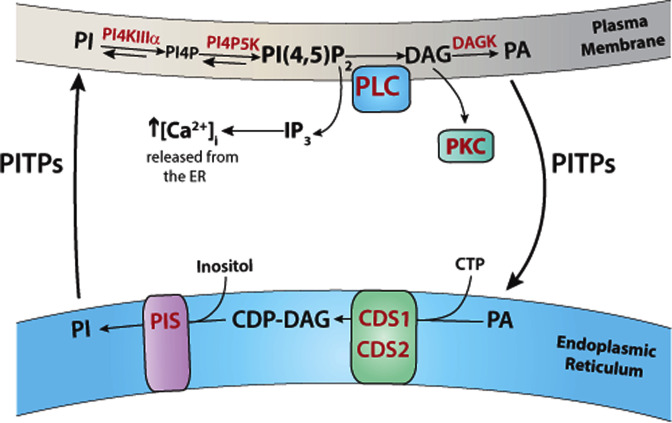
Synthesis and degradation of PI(4,5)P_2_ – phospholipase C cycle PLC hydrolyses PI(4,5)P_2_ resulting in the formation of the second messengers, IP_3_ and DAG. DAG is phosphorylated to PA at the plasma membrane by DAG kinase (DAGK). PA is transferred to the ER via lipid transfer proteins. In the ER, PA is converted into CDP-DAG catalysed by CDS enzymes (CDS1 and CDS2). In the final step, inositol and CDP-DAG are synthesised into PI catalysed by the enzyme, PI synthase (PIS). The newly synthesised PI is transferred to the plasma membrane for phosphorylation to PI(4,5)P_2_ by the resident enzymes, PI4KIIIα and PIP5K. Abbreviations: CDP-DAG, cytidine diphosphate diacylglycerol; CDS, CDP-DAG synthase; IP_3_, inositol(1,4,5)triphosphate; PA, phosphatidic acid; PI, phosphatidylinositol; PIS, PI synthase; PITP, phosphatidylinositol transfer protein; PI4P, PI 4-phosphate; PI(4,5)P_2_, phosphatidylinositol (4,5) bisphosphate.

PI(4,5)P_2_ is synthesised from PI at the plasma membrane by sequential phosphorylation by two lipid kinases, PI 4-kinase (PI4K) and PI4P5K (Figure 2). The first enzyme PI4K converts PI into PI4P. There are altogether four PI4K in the mammalian genome, Type II (α and β) and Type III PI4K (α and β) (there is no Type I PI4Ks as they were subsequently discovered to be PI 3-kinases). Of the four enzymes, PI4K Type IIIα (PI4KIIIα) plays a major role in the generation of PI4P at the plasma membrane [19,20]. PI4KIIIα is present in a complex with two adapter proteins, TTC7 (tetratricopeptide repeat domain 7) and EFR3 (protein encoded by the *EFR3* gene) that allows targeting to the plasma membrane [19]. The conversion of PI4P into PI(4,5)P_2_ is catalysed by PI4P 5-kinases and three isoforms (PI4P5Kα, β, γ) have been identified in mammals. However, the relative roles of each of the three PI4P5Ks remain to be characterised. PIP5Kγ is essential as mice lacking this enzyme do not survive [21].

Although PI phosphorylation to PI(4,5)P_2_ takes place at the plasma membrane, the synthesis of PI takes place in the ER [6]. PI synthesis is a two-step process, the conversion of PA (phosphatidic acid) into the intermediate, cytidine diphosphate DAG (CDP-DAG) by CDP-DAG synthase (CDS) enzymes followed by its conversion into PI by PI synthase (PIS) (Figure 2). There are two CDS enzymes, CDS1 and CDS2, and one PIS enzyme, both localised at the ER; all three enzymes are integral membrane proteins [6,22]. The first step requires CTP and the second-step requires inositol. PA can be either obtained by *de novo* synthesis, or from the PI(4,5)P_2_-PLC cycle. Following PLC activation, DAG is rapidly converted into PA and is utilised for the synthesis of PI (Figure 2). Due to the topological arrangement of the enzymes present in separate membrane compartments (i.e. plasma membrane and ER), lipid transfer of PI and PA has to take place. This is accomplished by a family of PI transfer proteins (PITPs) [23–26].

## PLC signalling

### PLC families, their regulation and biological functions

**Figure 3:**
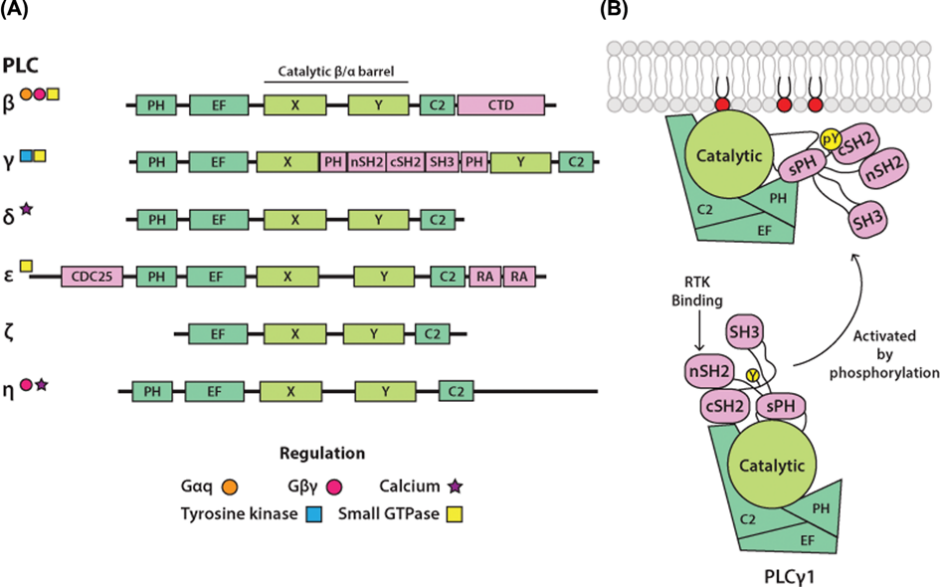
Mammalian phosphoinositide-specific phospholipase C (PLC) families (**A**) **Domain organisation of PLC enzymes**. Domain organisation of PLC families, showing the PLC-core (green), that includes the catalytic βα−barrel domain (light green), and domains unique for each PLC family (pink). Some of the well-characterised regulatory interactions are indicated by symbols. Abbreviations: CDC25, cell cycle division 25 (Ras GEF domain); cSH2, C-terminal SH2; CTD; C-terminal domain; C2, protein kinase C conserved region 2; EF, EF-hands; nSH2, N-terminal SH2; PH, pleckstrin homology domain; RA, Ras-association domain; SH2, Src homology 2 domain; SH3, Src homology 3 domain; sPH, split PH; X and Y, conserved halves of the catalytic domain. (**B**) **Mechanism of PLC activation**. One common aspect of PLC activation involves the release of autoinhibition. In PLCγ enzymes, the activation is triggered by phosphorylation of a specific Tyrosine (Y) residue (yellow) within the regulatory region. In the inactive form, two domains within the regulatory region (cSH2 and sPH) directly contribute to autoinhibition. Following phosphorylation, the critical pY residue (yellow) binds to the cSH2 domain resulting in repositioning of the regulatory region and release of autoinhibition.

PLCs hydrolyse different glycerophospholipids, including phosphoinositides, at the phosphodiester bond (between the glycerol backbone and the phosphate group). In mammals, PLC enzymes that use phosphoinositides (preferentially PI(4,5)P_2_) as their substrates have been grouped into six families (β, γ, δ, ε, ζ and η). Within each family are multiple members: four PLCβ (1–4), two PLCγ (1 and 2), three PLCδ (1, 3, 4), one PLCε, one PLCζ and two PLCη (1 and 2) making thirteen PLCs in total (reviewed in [27–33]) (Figure 3A). Recently, a seventh family of PLCs was discovered across different eukaryotic species, including three isoforms in humans, and named PLC-XD (PLC X-domain containing protein) [34]; more research is, however, needed to fully understand distinct properties and biological functions of PLC-XD enzymes. The PLC-XD enzymes are more related to bacterial PLCs whose substrate is PI rather than PI(4,5)P_2_ [35].

As outlined in Figure 3A, six PLC families share a conserved core structure in addition to a variety of other domains specific for each family. The conserved core structure comprises a PH, EF hands (helix–loop–helix structural domain found in Ca^2+^-binding proteins), X and Y and a C2 domain (protein kinase C conserved region 2; coloured green in Figure 3A). The enzyme activity in PLCs is encapsulated in the βα-barrel structure (the TIM-barrel (triosephosphate isomerase barrel) domain); X and Y correspond to the two halves of the barrel. Some of the regulatory elements are present in the common PLC-core domains as distinct features in different PLCs; for example, the PH domain (pleckstrin homology domain) in PLCδ1 binds PI(4,5)P_2_ while in PLCβ isoforms, it interacts with a small GTPase Rac. The regulatory function of many family-specific domains has been defined. In PLCβ, the unique C-terminal domain has been implicated in interactions with Gαq and with the membrane. In PLCγ isoforms, the linker between the two halves of the catalytic TIM-barrel differs from a relatively short, disordered region in all other families and is known as the γ-specific array (γSA) (coloured pink in Figure 3A). The γSA contains a ‘split’ PH domain (sPH), two Src homology 2 domains (nSH2 and cSH2) and a Src homology 3 (SH3) domain. The well-defined contacts with some members of the receptor tyrosine kinases (RTKs) and a small GTPase Rac, are examples of many regulatory interactions mediated by the γSA. PLCε contains a CDC25 domain (cell division cycle 25 (Ras GEF domain) has Ras GEF (guanine nucleotide exchange factor) activity) and two Ras association (RA) domains, both related to the regulatory interplay with small GTPases.

Together, the regulatory interactions embedded in the PLC-core and contained within the additional domains, provide links with numerous and diverse cell surface receptors [27–33]. Overall, the signalling connectivity remains best defined for the G-protein coupled receptors (GPCRs) and PLCβ isoforms, mediated by the α and βγ subunits of G-proteins, and for the RTKs and tyrosine kinases linked to immunoreceptor tyrosine-based activation motif (ITAM)-associated receptors, that activate PLC enzymes by direct phosphorylation. The regulation that involves small GTPases, activated by a range of different receptors, is also documented for several PLC families (PLCβ, PLCγ and PLCε) but the understanding of signalling links within relevant physiological contexts requires further studies. The importance of changes in cytosol Ca^2+^, in particular for the regulation PLCδ and PLCη isoforms, has also been suggested; however, precise binding sites on these PLCs are not clearly determined.

The presence of multiple PLCs with distinct regulatory links provides differential means of regulation of PLC activity, reflected in great diversity of their biological functions; this is illustrated here by several examples. Among many roles, ubiquitously expressed PLCβ1 enzyme has been implicated in control of neuronal function and the enhancement of glucose-stimulated insulin secretion in pancreatic β-cells downstream of specific GPCRs in these different cell types [36–40]. PLCγ2, highly expressed in hematopoietic cells, has the key role in signalling downstream of ITAM-associated receptors; for example, it controls multiple functions of B cells, and several types of innate immune cells in response to stimulation of the B-cell antigen receptor (BCR) and Fc receptors (FcRs), respectively [41,42]. Another illustration from a wide spectrum of different biological functions is provided by PLCζ1. This PLC is sperm-specific and is the physiological trigger responsible for generating I(1,4,5)P_3_-mediated Ca^2+^ oscillations that induces oocyte activation during mammalian fertilisation [43,44].

A substantial number of 3D structures for PLC enzymes provide a valuable basis for the understanding of various functional properties at the molecular level, including their PLC activity and regulatory mechanisms [45–49]. Notably, despite the diversity of their interacting proteins, the general molecular mechanism for regulation of PLCs is centred on intramolecular interactions that maintain PLCs in their inactive form, also referred to as autoinhibition, that becomes released in the process of activation. One example that illustrates this concept is provided by recent structural insights into PLCγ1, primarily regulated by RTKs (Figure 3B). In the inactive form, two domains within the regulatory region (cSH2 and sPH) directly contribute to autoinhibition by interacting with the PLC-core, preventing membrane interactions required for the access to the PLC substrate, PI(4,5)P_2_ [47,48]. Following phosphorylation of PLCγ1, the critical pTyr residue in PLCγ1 binds to its cSH2 domain; this intramolecular interaction is required for repositioning of the regulatory region and release of the autoinhibition.

### Downstream signalling

It is well established that both products of PLC hydrolysis, I(1,4,5)P_3_ and DAG, are second messengers. They regulate a range of functions by engaging ever-increasing number of protein targets and also through their further conversion by metabolic enzymes. I(1,4,5)P_3_ binds to IP_3_ receptors present at the ER to release Ca^2+^ into the cytosol from the ER stores whilst hydrophobic DAG binds to C1 domains (protein kinase C conserved region 1) of proteins for membrane recruitment and activation. I(1,4,5)P_3_ is also a substrate for the synthesis of inositol polyphosphates including pyro-phosphates such as IP_7_ and IP_8_ which are recognised as signalling molecules, including metabolic messengers or energy sensors [50]. Members of the PKC and Munc13 (mammalian uncoordinated-13) family as well as RasGRP4 (Ras guanyl-releasing protein 4) are prime examples of proteins that are regulated by transient changes in DAG [51–53]. In principle, conversion of DAG into PA also generates a bioactive metabolite with multiple functions [54–58]. PA can recruit and/or activate specific proteins such as PIP5K [55,56] and, with its cone-shaped geometry, PA can locally influence membrane topology and thus impact in membrane trafficking events [59]. However, it is more likely that the PA, generated during the PI(4,5)P_2_ – PLC cycle, is segregated for resynthesis into PI.

In addition to generation of second messengers, PI(4,5)P_2_ hydrolysis by PLC can decrease the levels of PI(4,5)P_2_. As already outlined in the introduction, PI(4,5)P_2_ concentrations regulate a number of processes by affecting recruitment of peripheral membrane proteins and by regulation of integral membrane proteins. Some specific examples, where changes in the PI(4,5)P_2_ levels caused by PLC activation regulate these processes, are provided in later sections.

## PI3K signalling

### Class I PI3Ks, their regulation and biological functions

PI3Ks phosphorylate the 3-hydroxyl group of the inositol ring in phosphatidylinositol lipids, allowing these to serve as ligands and functional regulators of a broad range of proteins. The three classes (Classes I, II and III) of these enzymes differ in their substrate specificity; the Class I PI3Ks selectively recognises and phosphorylates PI(4,5)P_2_ (reviewed in [60–63]).

**Figure 4:**
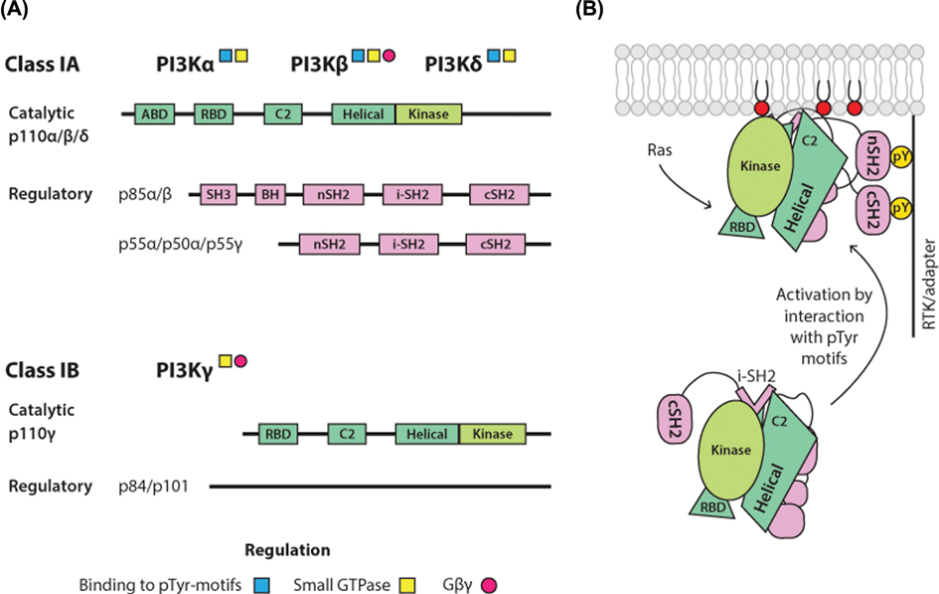
Class I Phosphoinositide-3-kinases (PI3K) (**A**) **Domain organisation of Class I PI3K**. Domain organisation of Class I PI3Ks (IA and IB) showing the catalytic subunits (green), that include the kinase domain (light green), and regulatory subunits (pink). Heterodimers, comprising a specific combination of one catalytic and one regulatory subunit within each subclass, are commonly designated based on the identity of the catalytic subunit as PI3Kα, PI3Kβ, PI3Kγ and PI3Kδ. Abbreviations: ABD, adaptor-binding domain; BH, breakpoint cluster region homology; cSH2, C-terminal SH2; C2, protein kinase C conserved region 2; i-SH2, inter-SH2 domain; nSH2, N-terminal SH2. (**B**) **Activation of PI3Kα**. Schematic of the activation of PI3Kα (p110α/p85α heterodimer) downstream of RTKs and adaptors containing phosphorylated YXXM-motifs (pYXXM). The binding of PI3Kα to these proteins at the membrane proximity is mediated by the SH2 domains in p85α, resulting in disruption of inhibitory contacts with the p110α catalytic subunits. Ras also activates PI3Kα, with Ras activation being strongly synergistic with activation downstream of phosphorylated RTKs and adapters.

The Class I enzymes act in signalling downstream of plasma membrane-bound receptors and the small GTPases. These PI3Ks are heterodimers of a p110 catalytic subunit (that includes the kinase domain) with a regulatory subunit that keeps the heterodimer in an inactive, cytosolic state. Mammals express four catalytic subunits (p110α, p110β, p110γ and p110δ) and five regulatory subunits (p85α, p85β, p55α, p50α and p55γ). A Class IA (p110α, p110β, and p110δ) binds the p85/p50/p55 type of regulatory subunits while Class IB (p110γ) binds one of two related regulatory subunits, p101 and p87, which have no homology to other proteins or recognisable domain structure (Figure 4A). Various domains that affect the kinase activity are present in both, catalytic and regulatory subunits of different isoforms and include the Ras-binding domain (RBD) that interacts with members of the Ras GTPase superfamily (the Ras and Rho families), SH2 domains that bind to phospho-tyrosine residues (pYXXM motifs) on growth factor receptors or adaptor proteins and a domain involved in binding to βγ subunits of heterotrimeric G proteins. As a generalised overview, activation of the lipid kinase present in p110α and p110δ is mediated by binding of their heterodimers to the pYXXM motifs, in p110γ through the binding of βγ subunits while p110β can be activated via both types of interactions. Additionally, all p110 catalytic subunits can interact with members of Ras GTPase superfamily. Notably, synergistic activation of specific Class I PI3K isoforms through different signalling inputs is an important aspect of their regulation [64].

In addition to differences in regulation, physiological roles of specific heterodimers are determined by their expression patterns and levels of expression. p110α and p110β have a broad tissue distribution. p110α heterodimers play a key role in glucose homeostasis and in insulin and growth factor signalling [65–67]. p110γ and p110δ are highly expressed in the immune cells but are also found in some other tissues at lower levels. They both play important, non-redundant roles in the immune system [68–70]. In addition to their diverse functions established in normal cells, PI3Ks are also quite extensively studied as targets for cancer therapy; the PI3K pathway is one of the most frequently dysregulated in cancer [71,72]. In particular, oncogenic mutations in the gene encoding the p110α catalytic subunit, *PIK3CA*, occur with high frequency in several common cancers [73].

Structural and biophysical studies have defined the mechanisms of autoinhibition and activation of different Class I isoforms. As a well-studied example, the p110α–p85 heterodimer and its activation by physiological signals is depicted in Figure 4B. In this case, the PI3K activity is inhibited by a combination of intra- and inter-subunit contacts that become disrupted following the engagement of the SH2 domains, present in p85, with the phosphorylated tyrosine residues in RTK/adapter proteins in stimulated cells; the activation also favours the interaction with the plasma membrane [74–76]. Interestingly, a number of frequent cancer mutations in *PIK3CA* upregulate the PI3K activity by mimicking or enhancing one or more conformational events that accompany the physiological activation [77].

### Effectors of PI(3,4,5)P_3_

The key to the understanding of PI3K signalling is the connectivity with downstream effectors of PI(3,4,5)P_3_. In addition to PI(3,4,5)P_3_ itself, its derivative PI(3,4)P_2_ (the product of dephosphorylation on the 5-position by the SHIP family of phosphatases (Src homology (SH2) containing inositol polyphosphate 5-phosphatase)) is recognised by a number of these effectors. PI(3,4,5)P_3_ and PI(3,4)P_2_ interact with the lipid-binding PH-domain in a range of protein effectors, resulting in their recruitment to membrane-signalling complexes and/or modulation of their activity [78–81]. Many Class I PI3K protein effectors bind to both PI(3,4,5)P_3_ and PI(3,4)P_2_. Interactions of proteins with these lipids, not mediated by the PH-domains or related modules, have also been described; one example are specific isoforms of the myosin motor proteins [82,83].

The PH-domain containing effectors comprise several subsets with common enzymatic or signalling functions. These include serine/threonine kinases such as AKT/PKB (protein kinase B), tyrosine kinases of the TEC (tyrosine kinase expressed in hepatocellular carcinoma) family particularly relevant for immune cells, modulators of small GTPase activities (various GEFs and GAPs (GTPase activating protein)) and scaffolding proteins (such as GAB (Grb2 (growth factor receptor bound protein 2)-associated binder) proteins). As a result, the activation of Class I PI3Ks can simultaneously trigger multiple, diverging downstream pathways. Compared with other effectors, the AKT kinases (AKT1, AKT2, AKT3) seem to be activated more universally downstream of receptor-mediated PI3K activation (reviewed in [81,84]). Following the PI(3,4,5)P_3_/PI(3,4)P_2_ binding by the PH domain and translocation to the membrane, AKTs undergo phosphorylation on two conserved residues (Thr^308^ by PDK1 and Ser^473^ by mTORC2), leading to their activation. More than 100 AKT substrates have been identified, including TSC2 (tuberous sclerosis complex 2 (also known as tuberin)) with the GAP function for a small GTPase RHEB (Ras homologue enriched in brain) and a number of FOXO (Forkhead family) transcription factors. The functional outcomes of TSC2 phosphorylation by AKT are well defined and linked to regulation of mTORC1 (mammalian target of rapamycin complex 1) by growth factor stimulation. As the key signalling node that coordinates anabolic metabolism and cell mass accumulation, mTORC1 integrates signals from nutrient availability with those from the growth factor receptors/Class I PI3Ks/AKT/TSC2 pathway. In contrast, the involvement of the FOXO transcription factors in PI3K signalling is less clear and most likely, substantially cell-context dependent; in T cells, the AKT/FOXO signalling controls cell differentiation and adaptation to nutrients and stress [85,86].

## Intact PI(4,5)P_2_ regulates actin cytoskeleton remodelling

**Figure 5:**
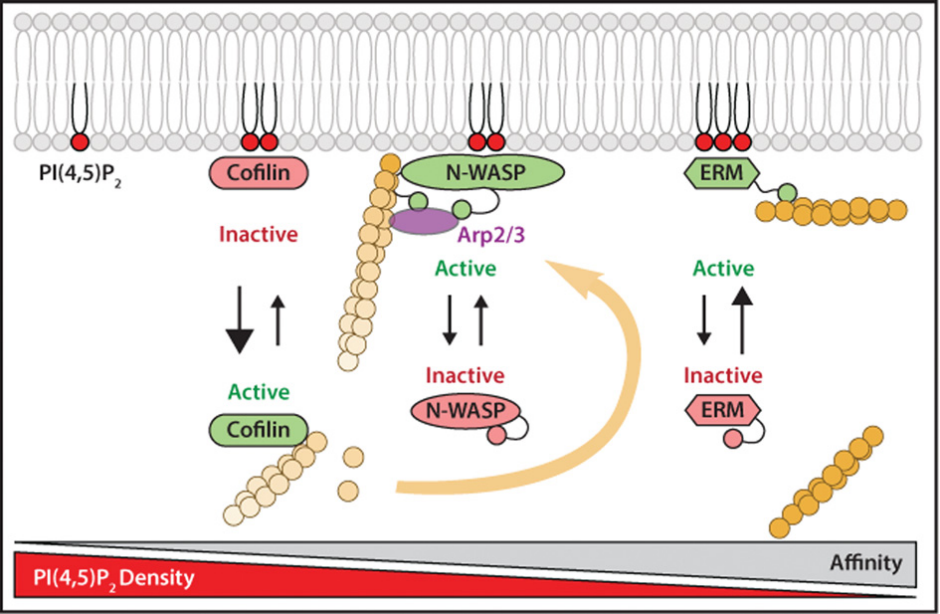
Actin cytoskeleton dynamics regulated by PI(4,5)P_2_ Regulation of the actin-binding proteins, cofilin, N-WASP and ERM proteins by PI(4,5)P_2_ levels. All these actin-binding proteins associate with PI(4,5)P_2_ through similar multivalent electrostatic interactions, but have different affinities for P(4,5)P_2_. Cofilin has low affinity, N-WASP has medium affinity and ERM proteins have high affinity. Cofilin is only bound to the membrane when PI(4,5)P_2_ is present at high density. When PI(4,5)P_2_ levels fall, cofilin is released into the cytosol to promote actin filament disassembly. In contrast, N-WASP interactions with PI(4,5)P_2_ results in a change in confirmation leading to activation; this allows the binding of actin-related protein 2/3 (Arp2/3) to mediate actin filament nucleation at the plasma membrane. ERM proteins are stably attached to the membrane by PI(4,5)P_2_ and link actin filaments to the plasma membrane. Cofilin and N-WASP require high PI(4,5)P_2_ density for interactions with the membrane, whereas ERM remain bound to the membrane at low PI(4,5)P_2_ density. Figure is adapted from [14]. Abbreviations: Arp2/2, actin-related protein 2/3; ERM, Ezrin, Radixin, Moesin; N-WASP, neural Wiskcott–Aldrich syndrome protein.

As illustrated for PI(3,4,5)P_3_ above, PI(4,5)P_2_ similarly binds and regulates a range of proteins; a subset of these downstream effectors is involved in regulation of actin cytoskeleton. Remodelling of the actin cytoskeleton occurs during many processes including cytokinesis, phagocytosis, endocytosis, cell motility and at focal adhesions. One of the main drivers for this process is PI(4,5)P_2_ [87]; it interacts with several actin-binding proteins at the plasma membrane, serving to regulate their activity through its levels (Figure 5) [14,88]. The actin cytoskeleton provides rigidity to the cells and is attached to the plasma membrane by Ezrin, Radixin and Moesin, collectively known as ERM proteins [89,90]. ERM proteins contain the FERM domain that directly binds to PI(4,5)P_2_. This interaction is important for releasing the autoinhibited state of the protein. ERM family proteins serve to securely cross-link actin filaments to the cell cortex; they have a very high affinity for PI(4,5)P_2_ and only dissociates from the membrane under extreme circumstances [14,91]. In lymphocytes, the chemokine, SDF-1 (stromal cell-derived factor 1 (also known as chemokine 12)), inactivates ERM proteins, causing their release from the plasma membrane following PLC activation [92]. Another class of linker protein between the plasma membrane and the actin cortex is class I myosin family proteins. Similar to the ERM proteins, class I myosins are also recruited to the membrane by PI(4,5)P_2_ [90].

In general, actin binding proteins have differing affinities for PI(4,5)P_2_, meaning that the level of PI(4,5)P_2_ at the plasma membrane can tightly regulate the dynamics of the actin cytoskeleton, with decreased PI(4,5)P_2_ levels having the overall effect of decreased actin stability. Overall, PI(4,5)P_2_ density plays an important role in cell motility by regulating the activity of actin binding proteins. Proteins such as cofilin that disassemble actin filaments have low affinity for PI(4,5)P_2_. Thus cofilin is retained at the plasma membrane under resting conditions when PI(4,5)P_2_ levels are high. During actin cytoskeletal remodelling, when PI(4,5)P_2_ levels are locally altered, proteins that aid actin filament disassembly such as cofilin are released into the cytosol where it can engage in disassembly of actin filaments making available actin monomers. Proteins such as N-WASP (neural Wiskott–Aldrich syndrome protein) that initiate actin polymerisation are active when interacting with PI(4,5)P_2_ [14,91]. N-WASP has a high affinity for PI(4,5)P_2_, and is activated in regions with a high PI(4,5)P_2_ density, which in turn activates the actin-related protein 2/3 (Arp2/3) complex to initiate actin nucleation. This is important for cell migration; N-WASP localise at extending lamellipodia which are regions of high PI(4,5)P_2_ density.

PLC activation results in decreased PI(4,5)P_2_ and this impacts on the actin cytoskeleton. For example, lamellipodial protrusion and directional migration of carcinoma cells towards chemoattractants, such as epidermal growth factor (EGF), depend upon the spatial and temporal regulation of the actin cytoskeleton. EGF induces a rapid loss of PI(4,5)P_2_ through PLC activity, resulting in release and activation of a membrane-bound pool of cofilin. Upon release, cofilin binds to and severs F-actin, which is coincident with actin polymerisation and lamellipodium formation [93].

Focal adhesions are structures that mechanically connect the extracellular matrix to intracellular actin bundles via integrins. Talin is an integrin-activating focal adhesion component directly connecting integrins in the plasma membrane with the actomyosin cytoskeleton [94,95]. Talin contains a FERM domain that allows the protein to attach to PI(4,5)P_2_. Talin also binds to PIP5Kγ, the enzyme that makes PI(4,5)P_2_, defining a mechanism for spatial generation of PI(4,5)P_2_ at focal adhesions [96–98].

## Endocytosis

**Figure 6:**
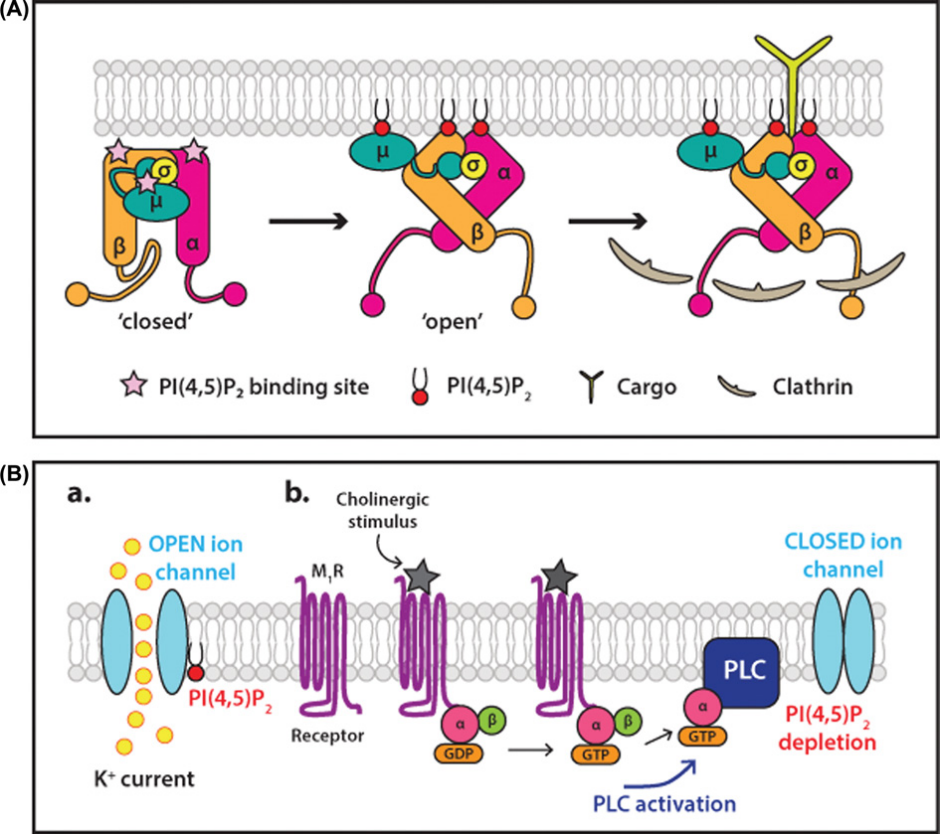
Examples of membrane peripheral and membrane integral proteins regulated by PI(4,5)P _2_ (**A**) **Binding of PI(4,5)P_2_ to the protein complex, AP2, changes its conformation to allow cargo and clathrin interactions**. The adaptor protein, AP2 is a complex of four proteins consisting of a core comprising the N-terminal domains of the α-and β2-adaptins in complex with the μ2 and σ2 subunits. The α, β2 and μ2 subunits all contain PI(4,5)P_2_ binding sites marked with pink stars. Long flexible linkers, referred to as hinge regions, connect the C-terminal appendage domains of α-and β2-adaptins to the core. AP2 exists in a closed conformation in the cytosol, in which the clathrin binding site is buried by interactions between the β2 hinge and the core and the cargo binding site on the μ2 subunit are also buried. Initially, the surface-exposed PI(4,5)P_2_ binding site on the α-and β2-adaptin interact with the lipid triggering an allosteric conformational change to an open conformation. This exposes the clathrin binding site on the β2 hinge as well as the PI(4,5)P_2_ and cargo binding sites of μ2. Figure adapted from [99]. (**B**) **Regulation of potassium channels by PI(4,5)P_2_ depletion by PLC**. Potassium channels are maintained in the open state when bound to PI(4,5)P_2_. Stimulation of the muscarinic M1 receptor by a cholinergic stimulus activates PLC to hydrolyse PI(4,5)P_2_. PI(4,5)P_2_ depletion results in closure of the ion channel. Abbreviations: M_1_R, M_1_ muscarinic receptor; PI(4,5)P_2_, phosphatidylinositol(4,5,)bisphosphate. Figure adapted from [154].

Internalisation of nutrients, cargo-bound receptors and ligand-bound signalling receptors takes place by clathrin-mediated endocytosis which requires PI(4,5)P_2_ [99]. PI(4,5)P_2_ at the plasma membrane localises the required endocytic machinery to the site of endocytosis. The adaptor protein, AP2 is a complex of four proteins consisting of a core comprising the N-terminal domains of the α-and β2-adaptins in complex with the μ2 and σ2 subunits. The α, β2 and μ2 subunits all contain PI(4,5)P_2_ binding sites. Long flexible linkers, referred to as hinge regions, connect the C-terminal appendage domains of α-and β2-adaptins to the core (Figure 6A). AP2 exists in a closed conformation in the cytosol, in which the clathrin binding site is buried by interactions between the β2 hinge and the core. The PI(4,5)P_2_ and cargo binding sites on the μ2 subunit are also buried in this conformation. The interaction of surface-exposed binding sites on both the α- and β2-adaptin with plasma membrane-enriched PI(4,5)P_2_ triggers an allosteric conformational change to an open conformation that exposes the clathrin binding site on the β2 hinge as well as the PI(4,5)P_2_ and cargo binding sites of μ2 (Figure 6A). The active conformation of AP2 can then recruit clathrin. A positive feedback loop is also established as AP2 activates PIP 5-kinase for increased PI(4,5)P_2_ production, promoting further recruitment of AP2 and assembly of endocytic vesicles [100].

Additional roles for PI(4,5)P_2_ are also central for completion of the endocytic process. After clathrin recruitment, PI(4,5)P_2_ facilitates membrane deformation. Epsin binds to PI(4,5)P_2_, localising epsin to the endocytic site where it inserts an amphipathic helix for membrane deformation [101,102]. Accessory proteins with BAR (domain named after three proteins: Bin, Amphiphysin and Rvs that share the domain) domains, which also bind PI(4,5)P_2_, also contribute to deformation of the membrane [103]. PI(4,5)P_2_ also plays a crucial role in the recruitment of dynamin to the plasma membrane where it assembles at the neck of the budding vesicle and mediates fusion of the non-cytosolic leaflets of the membrane [99]. PI(4,5)P_2_ is dephosphorylated by 5-phosphatases for uncoating to take place [104]. Thus, although PI(4,5)P_2_ facilitates the mechanism of clathrin vesicle endocytosis, excess PI(4,5)P_2_ inhibits endocytosis. Persistence of PI(4,5)P_2_ on vesicular membranes prevents the uncoating of the vesicle and subsequent vesicular fusion with the target membrane [105].

## Exocytosis

A potential role for phosphoinositides in exocytosis was first described by studies that used a bacterial PLC for depletion resulting in inhibition of Ca^2+^-mediated exocytosis in permeabilised chromaffin cells [106]. Subsequent work in several types of secretory cells found PI(4,5P)_2_ necessary for exocytosis [107,108]. Several PI(4,5P)_2_-binding proteins have been identified with important functions in SNARE complex assembly, including C2-domain-containing proteins, synaptotagmin-1 and Munc13-1 [109], PH- and C2-domain-containing protein CAPS (Ca^2+^-dependent activator protein for secretion), and syntaxin-1 [110]. Synaptotagmin-1 is a synaptic vesicle-associated membrane protein whilst Munc13-1 and CAPS are cytosolic protein recruited by PI(4,5)P_2_. In contrast syntaxin 1 is clustered by high concentration of PI(4,5)P_2_ at the plasma membrane. Thus PI(4,5)P_2_ participates in multiple aspects of exocytosis including docking, priming and fusion of secretory granules [111].

## Ion channel regulation by PI(4,5)P_2_

Like endocytosis, many ion channels and transporters in the plasma membrane also depend on the presence of PI(4,5)P_2_ for correct functioning [112–114]. PI(4,5)P_2_ acts directly on ion channels including inwardly rectifying K^+^ (Kir) channels, KCNQ (also known as Kv) channels and transporters such as the Na^+^/Ca^2+^ exchanger to facilitate their opening. This dependence on PI(4,5)P_2_ allows the activity of channels and transporters to be directly linked to cellular signalling. A variety of signalling pathways involve PLC activation and so PI(4,5)P_2_ depletion, leading to the inactivation of these PI(4,5)P_2_-dependent channels. The best characterised example is the KCNQ channels which are maintained in the open state allowing K^+^ to move freely (Figure 6B). Upon stimulation with the muscarinic agonist, M1 receptors are activated which couple to Gαq and activate PLCβ1. A robust decrease in PI(4,5)P_2_ causes channel closure; the PI(4,5)P_2_ hydrolysis products IP_3_ and DAG do not contribute directly to channel regulation. Resynthesis of PI(4,5)P_2_ is a rapid process which reopens the channels [115,116]. Another example is the Kir2.2 channel, which is maintained in the open state to allow inflow of K^+^. A crystal structure of the inward rectifier Kir2.2 channel shows that each subunit directly coordinates a single PI(4,5)P_2_ molecule in a conserved basic pocket to keep the channel open [12,13].

Regulation of ion channels by PI(4,5)P_2_ can either maintain channels in the ‘open’ or ‘closed’ state. The Ca^2+/^Na^+^ TRPV4 (transient receptor potential vanilloid 4) channel is inhibited by PI(4,5)P_2_ and opens when PI(4,5)P_2_ levels drop, the opposite to Kir2.1 channels. The depletion of PI(4,5)P_2_ by agonists such as prostaglandin E_2_, ATP or acetylcholine that signal through Gαq-PLCβ1 can therefore cause a simultaneous closure of Kir2.1 channels and the opening of TRPV4 channels as observed in endothelial cells [117].

A recent development is the use of high resolution cryo-electron microscopy to study structures of ion channels which are functionally reconstituted in lipid nanodiscs. The GABA_A_ receptor is a pentamer and two molecules of PI(4,5)P_2_ are constitutively associated with the receptor. The negatively charged headgroup of PI(4,5)P_2_ occupies a positively charged pocket in the intracellular juxta-membrane region of one of the subunits. The function of PI(4,5)P_2_ is not to regulate channel function. It is speculated that in a physiological context, this interaction may serve to sequester the protein to specific lipid microdomains, where trafficking the protein can be precisely regulated [13,118].

## PI(4,5)P_2_ stabilises interactions between GPCRs and Gα subunits and with arrestin

Recent studies highlight a role for PI(4,5)P_2_ in stabilising interactions between GPCRs and their binding partners, G-proteins and arrestins. PI(4,5)P_2_ binds to GPCRs such as the β1-adrenergic receptor, the adenosine A2 receptor, and the neurotensin receptor 1. The head group of PI(4,5)P_2_ specifically bridges the Gαs (but not Gαi or Gα12) subunit and the transmembrane domain of the β1-adrenergic receptor stabilising the active state of the GPCR [119]. Stabilisation of the receptors in the active state increases GTPase activity and enhances selectivity of coupling to G proteins.

To terminate GPCR signalling, the receptors are phosphorylated by G-protein receptor kinases (GRKs) promoting the binding of arrestin. This prevents G-protein coupling, triggering receptor internalisation and affecting various downstream pathways. The structure of the phosphorylated human neurotensin receptor 1 with arrestin reveals a PI(4,5)P_2_ molecule forming a bridge between the receptor and arrestin [120,121].

## Organisation of PI(4,5)P_2_ at the plasma membrane

As described above, many processes that require PI(4,5)P_2_ operate simultaneously at the plasma membrane. This raises the question of how the different requirements of PI(4,5)P_2_-dependent functions are maintained. Our understanding of the plasma membrane has evolved with the recognition that the lipids are not homogeneously distributed but are segregated; one early concept was ‘lipid rafts’ as platforms enriched in cholesterol and sphingolipids, in which specific proteins involved in signalling can accumulate [122,123].

PI(4,5)P_2_ segregation has been studied by comparing its diffusion at the cytoplasmic leaflet of cellular plasma membranes and membranes devoid of protein [124]. The diffusion coefficient is much lower and results indicate that two thirds of the PI(4,5)P_2_ is reversibly bound to proteins. Similar results have been seen in red blood cells where 50% of the PI(4,5)P_2_ is bound to cytoskeletal proteins [125]. A further refinement of this concept is the formation of dynamic clusters of PI(4,5)P_2_ at nanoscale. Using super-resolution stimulated-emission depletion (STED) microscopy on the plasma membranes of PC12 cells, PI(4,5)P_2_ was found in clusters of ∼65–73 nm in size [110,126]. Basically, current studies strongly suggest that PI(4,5)P_2_ clusters in the cytoplasmic leaflet align with cholesterol- and sphingomyelin-rich regions in the external leaflet of the plasma membranes by a mechanism referred to as trans-bilayer coupling [111,127–130]. Local enrichment of PI(4,5)P_2_ can occur by multiple mechanisms [131,132]. There can be preferential trapping of PI(4,5)P_2_ in lipid rafts, binding proteins such as MARCKS (myristoylated alanine-rich C-kinase substrate), syntaxin-1 and K-Ras that sequester PI(4,5)P_2_, or localised recruitment of PIP5K to generate PI(4,5)P_2_. Although there is strong evidence to support segregation of PI(4,5)P_2_, as discussed above, there remains many caveats due to technical limitations [132,133].

## Future directions

The present and past decades have seen a tremendous surge in the study of phosphoinositide signalling and reiterated their important place in regulation of diverse biological processes; the list continues to increase to span many cellular functions and their dysregulation in disease. Among different phosphoinositides, PI(4,5)P_2_ has an important role both, as a substrate for two types of key signalling enzymes (PLC and PI3K) and as a regulatory ligand for peripheral and integral membrane proteins. Many important proteins in different signalling networks linked to PI(4,5)P_2_ have been extensively characterised. However, further structural and functional characterisation of higher order complexes and more detailed insights into allosteric regulation of proteins by the PI(4,5)P_2_-binding (particularly relevant for ion channels and GPCRs) is needed; in pursuing these directions, we are likely to see an increasing contribution from methodologies such as cryo-EM. Although not covered in this review, the importance of aberrant functions of different PLCs and PI3Ks in disease development is well established and continues to expand [11,62,67,72,134–138]. Therefore, these efforts are likely to have a significant translational value, notably for drug discovery. The need for more cellular and physiological studies is also apparent. For example, as we understand more and more about the importance of spatial and temporal organisation and connectivity of the PI(4,5)P_2_ signals, it has become clear that we need to follow changes in live cells with subcellular resolution; the techniques capable of achieving super-resolution level imaging are likely to play an important contribution in this area. Some tools are available for specifically imaging PI(4,5)P_2_ (including the widely-used PH domain of PLCδ) but these have limitations and therefore further development is required.

## Summary

- PI(4,5)P_2_ plays many roles in the plasma membrane.
- PI(4,5)P_2_ is a substrate for two signalling pathways, PLC and PI3K.
- PI(4,5)P_2_ regulates many actin binding proteins for actin cytoskeleton dynamics.
- PI(4,5)P_2_ recruits many protein for endocytosis and for exocytosis.
- Ion channels and GPCRs are regulated by changes in PI(4,5)P_2_ levels that can be mediated by PLC.

## Open Access

Open access for this article was enabled by the participation of University College London in an all-inclusive *Read & Publish* pilot with Portland Press and the Biochemical Society under a transformative agreement with JISC.

## Abbreviations

AKT: serine/threonine kinase, also known as PKB
CAPS: Ca^2+^-dependent activator protein for secretion
CDC25 domain: cell division cycle 25 (Ras GEF domain)
CDP-DAG: cytidine diphosphate diacylglycerol
CDS: CDP-DAG synthase
DAG: diacylglycerol
ER: endoplasmic reticulum
FERM: domain found in four proteins Band 4.1, ezrin, radixin and moesin
FOXO: Forkhead family of transcription factor
GAP: GTPase activating protein
GEF: guanine nucleotide exchange factor
GPCR: G-protein coupled receptor
IP_3_/I(1,4,5)P_3_: inositol(1,4,5)trisphosphate
ITAM: immunoreceptor tyrosine-based activation motif
Kir: inward rectifying K^+^ channel
mTORC1: mammalian target of rapamycin complex 1
Munc13: mammalian uncoordinated-13
N-WASP: neural Wiskott–Aldrich syndrome protein
PA: phosphatidic acid
PDZ: domain named after three proteins: PSD95, Dig1 and Zo-1 that share the domain
PH domain: pleckstrin homology domain
PI: phosphatidylinositol
PI(3,4,5)P_3_: phosphatidylinositol(3,4,5)trisphosphate
PI(4,5)P_2_: phosphatidylinositol(4,5)bisphosphate
PI3K: phosphoinositide 3-kinase
PI4K: PI 4-kinase
PI4P: phosphatidylinositol 4-phosphate
PI4P5K: PI4P 5-kinase
PIS: PI synthase
PKC: protein kinase C
PLC: phospholipase C
PLC-XD: PLC X-domain containing protein
RA: Ras association
RTK: receptor tyrosine kinase
SH2: Src homology 2
SH3: Src homology 3
SNARE: Soluble N-ethylmaleimide-sensitive factor attachment protein receptors
TIM barrel: triosephosphate isomerase barrel
TRPV4: transient receptor potential vanilloid 4
TSC2: tuberous sclerosis complex 2 (also known as tuberin)
γSA: γ-specific array
